# A case of autism spectrum disorder with cleft lip and palate carrying a mutation in exon 8 of *AUTS2*

**DOI:** 10.1002/ccr3.2377

**Published:** 2019-09-24

**Authors:** Saki Saeki, Takashi Enokizono, Kazuo Imagawa, Hiroko Fukushima, Daigo Kajikawa, Aiko Sakai, Mai Tanaka, Tatsuyuki Ohto, Hisato Suzuki, Tomoko Uehara, Toshiki Takenouchi, Kosaki Kenjiro, Hidetoshi Takada

**Affiliations:** ^1^ Department of Pediatrics, Faculty of Medicine University of Tsukuba Tsukuba Japan; ^2^ Department of Child Health, Faculty of Medicine University of Tsukuba Tsukuba Japan; ^3^ Center for Medical Genetics Keio University School of Medicine Tokyo Japan

**Keywords:** autism spectrum disorder, AUTS2, cleft lip and palate, exon 8

## Abstract

We report a patient with autism and cleft lip and palate carrying a de novo heterozygous *AUTS2* mutation, c.1464_1467del ACTC (p.Tyr488*). Although the causal relationship between cleft lip and palate and this mutation is unclear, this case report may expand the clinical phenotype of AUTS2 syndrome.

## Graphical abstract

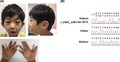

## 1 INTRODUCTION

Autism spectrum disorder (ASD) is characterized by impairments in ordinary social communication, along with restricted and stereotyped behaviors and interests. The morbidity rate remains under 1% of the population with onset before the age of 3 years. In recent years, advances in genomic techniques have provided novel insight into the genetic causes of ASD,^1^ including mutations in the autism susceptibility candidate 2 (*AUTS2*) gene.

*AUTS2* is a well‐conserved gene in vertebrates, comprising 19 exons located on chromosome 7q11.22. The frequency of *AUTS2* mutation is 1/2000.^2^, ^3^ Its 19 exons are divided into two parts: the first six exons at the 5′ end, which are separated by large introns, and the remaining 13 exons at the 3′ end, which are compact with smaller clustered introns.^4^ In addition to ASD, variants in *AUTS2* have been demonstrated to be associated with other neurodevelopmental disorders such as schizophrenia and developmental delay,^1^ along with physical malformations such as dysmorphic features, short stature, and microcephaly.^2^

We encountered a patient with ASD and a heterozygous *AUTS2* mutation that was defined as likely pathogenic in ClinVar (GenomeDx, RCV000657843.1). However, the phenotype of this mutation has never been reported.

Here, we report the clinical details of a patient with this mutation. To our knowledge, this is the first case of an *AUTS2* variant associated with cleft lip and palate.

## 2 CASE REPORT

**Figure 1:**
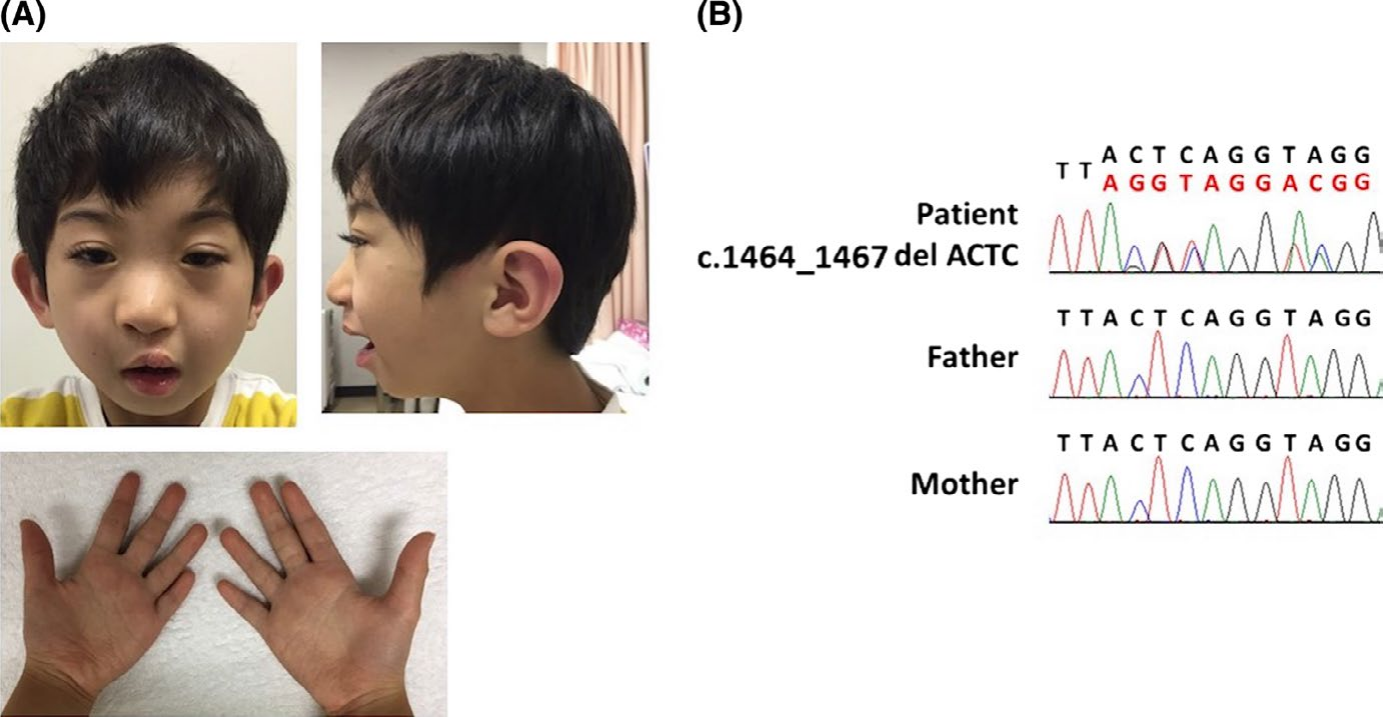
Characteristics of the patient carrying a mutation in exon 8 of *AUTS2. *A, Proband at the age of 8 y. These photographs show the highly arched eyebrows, broad nasal bridge, microcephaly, retrognathia, low‐set ears, narrow mouth, and short length of the fifth fingers but no skeletal anomaly. B, Sanger sequencing was performed, and a de novo mutation, c.1464_1467del ACTC (p.Tyr 488*), in *AUTS2* was identified in the proband

The 8‐year‐old patient was the first child born to nonconsanguineous, healthy Japanese parents. The family history was unremarkable. The boy was born at 36 weeks of gestation by vaginal delivery and did not suffer asphyxia. His birth weight was 2005 g (−2.5 SD), with a height of 44.2 cm (−2.3 SD), and head circumference of 26.5 cm (−4.8 SD). He exhibited dysmorphic features, including highly arched eyebrows, micrognathia (Figure 1A), cleft lip and palate, microcephaly, left cryptorchidism, right inguinal hernia, and a ventricular septal defect that was closed at 10 months of age. The cleft lip and palate were treated surgically at 10 and 19 months of age, respectively. His motor and intellectual developments were delayed from early childhood; he walked alone at 23 months old and spoke a single word at 35 months old. He required tube feeding from the age of 4 months owing to feeding difficulties. Brain magnetic resonance imaging and electroencephalogram revealed no abnormalities. Various blood tests, including amino acid analysis, lactate, and pyruvate, were normal. Chromosomal analysis and array comparative genomic hybridization showed no abnormalities.

At 3 years of age, the patient exhibited marked hyperactivity, impulsivity, and acoustic hyperesthesia. His face was always expressionless, and he could not make eye contact. He showed developmental language delay, and the diagnosis of ASD was finally made. Medications were needed to control his remarkable hyperactivity, such as risperidone, atomoxetine, and aripiprazole. Oral food intake gradually increased, and nasogastric tube feeding was not required by the age of 3 years and 9 months; however, his feeding difficulties continued until almost 5 years of age. He was still small in terms of physical status at 5 years and 10 months: weight 10.9 kg (−2.9 SD), height 94.6 cm (−3.7 SD), and head circumference 43.0 cm (−4.4 SD). He is currently enrolled in special support education classes.

A trio exome analysis was performed as described previously.^5^ The research protocol was approved by the local institutional ethical review board of the University of Tsukuba and Keio University School of Medicine following the Ethical Guidelines for Human Genome/Gene Analysis Research of the Ministry of Health, Labor and Welfare of Japan and the Declaration of Helsinki Principles. Informed consent was obtained from the parents. Genomic DNA was extracted from peripheral blood leukocytes of the patient and both parents, and whole‐exome sequencing was performed using the SureSelect XT Human All Exon V6 (Agilent Technologies) and HiSeq 2500 (Illumina) platforms. Whole‐exome sequencing revealed a mutation in autism susceptibility candidate 2 (*AUTS2*), NM_015570.3:c.1464_1467del ACTC (p.Tyr488*), which was determined to be a de novo mutation in the patient (Figure 1B). These sequence data have been reported to ClinVar by GenomeDx under accession number RCV000657843.1. This variant is located in exon 8, at the C‐terminus of *AUTS2* and is predicted to disrupt mRNA translation.

## 3 DISCUSSION

The main functions of *AUTS2* involve neuronal migration, extension, and branching of the neurites and construction of the correct neuronal network.^4^ Previous studies have indicated that mutations in *AUTS2* are associated with not only neurodevelopmental disorders such as ASDs, attention deficit hyperactivity disorder, and intellectual disability but also short stature, microcephaly, feeding difficulties, dysmorphic features, and other malformations.^7^, ^8^, ^9^ Beunders et al^2^ coined the term "AUTS2 syndrome" to describe these various symptoms and established a scoring system composed of four grades: 0‐7, 8‐12, 13‐18, and 19‐31. Patients with deletions at the N‐terminus typically show only neurodevelopment disorders, whereas those with deletions at the C‐terminus exhibit neurodevelopment disorders as well as other physical features and feeding difficulties.^1^, ^2^ Mutations in the C‐terminus of *AUTS2* appear to cause more severe phenotypes. Our patient has a deletion in exon 8 of *AUTS2*, which is located in the C‐terminus, and he presents a severe phenotype consistent with these previous descriptions. Indeed, the patient's AUTS2 syndrome score was 16/31, which is classified as the second most severe grade^2^ (Table 1).

**Table 1:** Features of the AUTS2 syndrome depending on region of the deletion^2^

Cases	Our case	Beunders' cases					
Exon deleted	8	1 to 5		6 to 19 (c terminal)		all	
Number of the patients	1	7		10		2	
Growth and feeding							
Low birth weight	+	1/7	14%	4/10	40%	1/2	50%
Short stature <p10^a^	+	1/7	14%	9/10	90%	1/2	50%
Microcephaly <p2^b^	+	3/7	43%	8/10	80%	1/2	50%
Feeding difficulties	+	1/7	14%	6/10	60%	1/2	50%
Neurodevelopmental disorders							
Intellectual disability	+	6/7	86%	10/10	100%	1/2	50%
Autism/autistic behavior	+	2/7	29%	3/10	30%	0/2	0%
Sound sensitivity	+	0/7	0%	0/10/	0%	2/2	100%
Hyperactivity/ADHD	+	0/7	0%	2/10	20%	1/2	50%
Other	−	1/7	14%	2/10	20%	0/2	0%
Neurological disorders							
General hypotonia	−	1/7	14%	4/10	40%	1/2	50%
Structural brain anomaly	−	1/7	14%	2/10	20%	0/2	0%
Cerebral palsy/spasticity	−	1/7	14%	6/10	60%	2/2	100%
Other	−	1/7	14%	2/10	20%	0/2	0%
Dysmorphic feature							
Highly arched eyebrows	+	1/7	14%	6/10	60%	0/2	0%
Hypertelorism	−	0/7	0%	7/10	70%	1/2	50%
Proptosis	−	1/7	14%	5/10	50%	0/2	0%
Short palpebral fissures	−	2/7	29%	3/10	30%	1/2	50%
Up slanting palpebral fissures	−	0/7	0%	1/10	10%	1/2	50%
Ptosis	−	2/7	29%	5/10	50%	0/2	0%
Epicanthal fold	−	1/7	14%	5/10	50%	0/2	0%
Strabismus	−	2/7	29%	3/10	30%	0/2	0%
Prominent nasal tip	−	0/7	0%	4/10	40%	0/2	0%
Anteverted nares	−	0/7	0%	2/10	20%	0/2	0%
Deep/broad nasal bridge	+	1/7	14%	3/10	30%	2/2	100%
Short/upturned philtrum	−	2/7	29%	4/10	40%	1/2	50%
Micro/retrognathia	+	1/7	14%	5/10	50%	1/2	50%
Low‐set ears	+	0/7	0%	5/10	50%	0/2	0%
Ear pit	−	0/7	0%	2/10	20%	0/2	0%
Narrow mouth	+	3/7	43%	6/10	60%	1/2	50%
Other	+	1/7	14%	5/10	50%	2/2	100%
Skeletal disorders							
Kyphosis/scoliosis	−	0/7	0%	2/10	20%	0/2	0%
Arthrogryposis/shallow palmar creases	−	0/7	0%	2/10	20%	0/2	0%
Tight heel cords	−	0/7	0%	2/10	20%	2/2	100%
Other	−	1/7	14%	3/10	30%	1/2	50%
Congenital malformations							
Hernia umbilicalis/inguinalis	+	0/7	0%	2/10	20%	0/2	0%
Patent foreman ovale/atrial septum defect	−	1/7	14%	1/10	10%	1/2	50%
Other	−	2/7	29%	3/10	30%	1/2	50%
Median AUTS2 syndrome severity score (range)	15	5 (0‐11)	12 (8‐22)	11 (7‐16)			

a Short stature is defined as height below the tenth percentile.

b Microcephaly is defined as skull size below the second percentile.

Considering the biological function of *AUTS2* in the brain, researchers found that nuclear AUTS2 combines with the promoter/enhancers of various genes related to brain development.^4^, ^10^ Gao et al^11^ showed that the most important amino acids to recruit P300, which is a well‐known histone acetyltransferase known to play a role in craniofacial development, may be located at positions 404‐717.^11^ These positions correspond to the region between exon 7 and exon 16.^2^ The de novo mutation identified in our patient creates a stop codon at amino acid 488 in exon 8. This mutation may cause nonsense‐mediated decay and loss of the entire cDNA or result in loss of the region needed for recruiting P300. Many distant‐acting enhancers for craniofacial development are also associated with P300.

The main unique features of our AUTS2 syndrome patient are the cleft lip and palate. The prevalence of cleft lip and palate is approximately 1.7 per 1000 live births in Asia, which also has the highest average occurrence rate for cleft lip and palate worldwide. In Japanese populations, the prevalence of cleft lip and palate is 2.06 per 1000 live births and shows a significant male bias.^6^ The definite cause of cleft lip and palate remains largely unknown.^6^ There are many known risk factors, including maternal smoking, alcohol consumption, medications, nutrition, and genetic factors. Our patient did not have any of these risk factors for cleft lip and palate except for ethnic group and sex.

Since this is the only reported case of cleft palate associated with *AUTS2* mutation, a causal relationship warrants further investigation. Moreover, further studies should focus on the broader functions of *AUTS2* beyond its role in the nervous system to explain why a variant of this gene would cause physical malformations, including cleft lip and palate.

## CONFLICT OF INTEREST

The authors have no conflict of interest to declare.

## AUTHOR CONTRIBUTIONS

SS, TE, MT, and TO examined the patient. HS, TU, TT, and KK performed exome analysis. KI, HF, DK, AS, MT, HS, TU, TT, and KK analyzed data. SS, TE, KI, and HT wrote the paper. All authors have read and approved the final manuscript.
